# Patient preferences for dry powder inhaler attributes in asthma and chronic obstructive pulmonary disease in France: a discrete choice experiment

**DOI:** 10.1186/s12890-017-0439-x

**Published:** 2017-07-06

**Authors:** Natalia Hawken, Saku Torvinen, Mohamed-Elmoctar Neine, Ikbel Amri, Mondher Toumi, Samuel Aballéa, Adam Plich, Nicolas Roche

**Affiliations:** 1 Creativ-Ceutical, Westblaak 92, 3012 KM Rotterdam, The Netherlands; 2 Teva Pharmaceuticals Europe BV, Amsterdam, The Netherlands; 3 Creativ-Ceutical SARL, 215, rue du Faubourg St-Honoré, 75008 Paris, France; 4 Creativ-Ceutical, Rue du lac Huron Résidence Farah, Bloc B, 1053 Les Berges du Lac, Tunisia; 5 Respiratory and Intensive Care Medicine, Cochin Hospital Group, AP-HP, University Paris Descartes, Paris, France

**Keywords:** Discrete choice experiment, Asthma, COPD, Patient preference, Willingness to pay

## Abstract

**Background:**

Dry powder inhalers (DPIs) are often used in asthma and chronic obstructive pulmonary disease (COPD) therapies. Using the discrete choice experiment (DCE) methodology, this study conducted in France was designed to assess patients’ preferences for different attributes of DPIs.

**Methods:**

Attributes of DPIs were defined based on a literature review, patient focus group discussions and interviews with healthcare professionals (qualitative phase of the study). An online survey was then conducted among French patients with asthma or COPD to elicit patient preferences and willingness to pay (WTP) for these attributes using the DCE methodology (quantitative phase). A fractional factorial design including three blocks of 12 choice sets was created. Each choice set comprised three alternatives: two fictitious inhalers and the patient’s current inhaler. Marginal utilities were estimated using a ranked ordered logit model. Interactions between attributes and disease (asthma or COPD) were tested.

**Results:**

Six DPI attributes were defined based on the qualitative phase: ease of use/fool-proof priming; accurate and easy-to-read dose counter; dose confirmation; hygiene of the mouthpiece; flexibility of the device handling; ability to use the inhaler with breathing difficulties. Overall, 201 patients with asthma and 93 with COPD were included in the online survey. Patients with asthma placed most value on an inhaler that requires one step for dose preparation (WTP €4.83 [95% CI: €3.77–€5.90], relative to an inhaler requiring four steps) and one that could be used during episodes of breathing difficulties (WTP €4.49 [95% CI: €2.95–€6.02]). Patients with COPD placed most value on an inhaler that could be used during episodes of breathing difficulties (WTP €7.70 [95% CI: €5.65–€9.76]) and on the accuracy of the dose counter (WTP €5.87 [95% CI: €3.98–€ 7.77]).

**Conclusion:**

This study suggests that asthma and COPD patients would be willing to change their inhaler if they were offered the option of a new inhaler with improved characteristics and they place a high value on an inhaler with ease of use during breathing difficulty episodes.

**Electronic supplementary material:**

The online version of this article (doi:10.1186/s12890-017-0439-x) contains supplementary material, which is available to authorized users.

## Background

Asthma and chronic obstructive pulmonary disease (COPD) are chronic airway diseases with significant socioeconomic burden. Inhalation therapy is the cornerstone of treatment for patients with asthma and COPD. A wide variety of different inhalers are available and can be broadly classified into pressurised metered-dose inhalers (pMDIs), dry powder inhalers (DPIs), breath-actuated metered-dose inhalers (BA-MDIs), and soft mist inhalers (SMI).

Although evidence from randomised controlled trials suggests that all inhaler types could be equally efficacious [1], each inhaler exhibits distinct operating characteristics and good inhaler technique is crucial for achieving optimal treatment effects [2, 3]. Studies have shown that inhaler technique remains poor and the majority of patients do not use their inhalers correctly [4, 5]. Additionally, poor inhaler use is associated with impaired symptom control in both asthma and COPD [5–7]. Taken together, outcomes from these studies suggested that the ideal inhaler is not currently available. Accordingly, novel inhalers continue to be developed to maximise use and minimise the possibility for incorrect inhaler technique.

Treatment compliance and therapeutic success of the inhalation therapy are dependent not only on the inhaler characteristics, but also on patients’ perception, satisfaction, and preference with the inhaler device [8, 9], as well as their competence in its use [5]. Suboptimal adherence is clearly associated with poor outcomes in patients with asthma and COPD [10]. Multiple factors drive patients’ preference with an inhaler; important features are the intuitiveness of the device and to ensure that correct inhaler technique only requires a limited number of steps, such as the sequence ‘exhale – open – inhale – close’ [11]. Therefore, the understanding of these factors is key to improving adherence to therapy. There is ongoing research to develop new DPIs for patients with asthma and COPD, highlighting the need to understand DPI attributes preferred by patients. Conjoint analyses and discrete choice experiments (DCEs) are frequently applied in healthcare to elicit preferences among patients [12, 13]. Respondents are asked to choose a preferred option from hypothetical scenarios designed to reflect the different attributes encompassed in real-world decisions. DCE can also be used to estimate willingness to pay (WTP) for healthcare interventions [12, 13]. This knowledge can assist healthcare professionals and health authorities in understanding the importance of developing new DPIs that are accessible to patients.

Using the DCE methodology, this study was designed to investigate patient preferences for attributes of DPI devices among patients with asthma or COPD treated with Symbicort® Turbuhaler® and Seretide® Diskus® in Europe. Here we report on findings among patients enrolled in the pilot phase of this study which was performed in France.

## Methods

The study comprised two phases. The first, qualitative phase followed a three-step process: (1) a literature review to identify the attributes for DPIs from previous studies; (2) clinical expert interviews; and (3) patient focus group discussions. The second, quantitative phase was the DCE.

### Definition of inhaler attributes

#### Literature review

A literature review on treatment satisfaction and drivers of adherence related to inhalation treatments among patients with asthma or COPD was conducted. This literature review aimed to identify attributes captured in studies evaluating treatment satisfaction among asthma and COPD patients using inhalers. Databases and keywords used for the literature search are listed in Additional file 1: Table S1. Attributes identified from the literature review formed the basis of discussion items among the patient groups. Most often cited inhaler characteristics are listed in Table 1.

**Table 1:** Most often used terms to describe the different concepts in literature

Concept	Times inhalers characteristics belonging to concept were mentioned in the literature search results	Examples of terms used in the literature to describe the concepts
Appearance	56	Attractiveness, design, colour, shape, size, compact, discrete, weight, portable and durable, protected from moisture
Ease of use, of which:	43	Operating, ease of inhalation, inhaler use quickness, handling, convenience
Ease of holding and comfort	11	Mouthpiece fits the lips, feel in the hand, easy to hold, ease of carrying
Ease of preparation of the dose	8	Ease to unpack and assemble, ease to open the inhaler and prepare it for inhalation
Feedback to indicate correct inhalation and dosage	16	Easily readable and precise dose counter
Ease of learning to use	14	Easy-to-follow instructions, easy-to-read leaflet, short training time required
Hygiene	10	Hygienic and easy to clean
Environmentally friendly	4	Inhaler and pack being environmentally friendly

#### Clinical expert interviews

Two French clinical practitioners who specialised in respiratory diseases were contacted through our network, and were interviewed using semi-directed or open questions to provide their expert opinion and rank the identified inhaler attributes from the literature searches. Topics discussed included: inhaler attributes that are important for correct inhaler technique, critical errors in inhaler technique (committed by the patients), and advantages and disadvantages of different inhalers (from the point of view of the clinician). The aim of the interviews was to gather information and develop a discussion guide for the focus group interviews.

#### Patient focus group discussions

A total of 32 patients identified through general practitioners and pharmacists in France with asthma (age range: 21–70 years) and COPD (age range: 43–82) who had used either a Symbicort Turbuhaler or Seretide Diskus, two of the most frequently used DPIs for inhaled corticosteroids/ long acting beta agonist, for ≥6 months prior to study were enrolled in four focus groups. Written informed consent was signed and dated by patients. Each focus group, consisting of patients with either asthma or COPD, was interviewed separately on a semi-structured basis to identify potential inhaler attributes. The discussions were moderated by an experienced psychologist. Discussions were structured in accordance with results of the literature search and clinician interviews.

#### Discrete choice experiment design

A pilot study was initially performed to identify any potential areas of misunderstanding. Twenty-five patients (10 with asthma, 10 with COPD and five with mixed COPD/asthma) participated in a cognitive debriefing interview and were asked to complete the survey in a ‘think aloud’ exercise. An additional 17 patients completed the pilot survey. An orthogonal and balanced fractional factorial (FrF) design was generated for the pilot study. This design contained 36 choice sets, divided into three blocks of 12, in order to reduce the number of questions for each participant. Each choice set consisted of three alternatives: two fictitious inhalers and the patient’s current inhaler. Inhalers were described according to attributes defined earlier, as well as a cost attribute. Minor rewording for some attributes was made based on the comments received during the pilot study. In addition, it was decided to present choices in two steps: first a choice between two fictitious inhalers, and then between the inhaler chosen in first step and the current inhaler.

The results from the pilot study were used to construct/develop the experimental design for the main survey, based on the D-efficiency criterion [14], assuming no interactions between attributes. The final design consisted of three blocks of 12 choice sets each.

WTP was defined by asking patients to respond to the following questions: (1) “What is your monthly out-of-pocket expenditure for the treatment of your asthma/COPD? Please take into account all costs directly or indirectly related to your treatment, such as prescription charges, travel costs to visit you doctor or go to the pharmacy, cost of taking time off work to go to the doctor”; (2) “Perhaps you also use healthcare services not covered by the NHS for the treatment of your asthma/COPD? Can you please estimate your monthly total out of pocket expenses (Euros/month)?”

### Experimental procedure

Patients ≥18 years of age with a diagnosis of asthma or COPD, and who were using a DPI (either Symbicort Turbuhaler or Seretide Diskus), were included in the study. Patients were recruited through an online research panel. To be eligible for participation, the panel members were asked to indicate the conditions with which they had been diagnosed by a physician, and those with asthma and COPD were selected. Moreover, there was no restriction with regard to the disease control status of patients to participate in this study. The DCE used a web-based platform that allowed participants to complete the survey at home. A screening question was used to ensure eligibility of those responding to the invitation. To help ensure that the correct inhalers were used for the study, patients were presented with pictures of Seretide Diskus and Symbicort Turbuhaler. Before making choices between inhaler profiles, respondents were presented with a description of their current inhaler and asked to state what they considered to be the three most important attributes of that inhaler. This was carried out to familiarise participants with the terminologies of inhaler attributes prior to performing the DCE exercise.

### Data collection

Demographic and baseline characteristics, medication history, and quality-of-life data were collected during the survey. Subjective health status was measured by health utilities index mark III (HUI3; a generic multi-attribute, preference-based measure of health status and health-related quality of life that is widely used as an outcome measure in clinical studies, population health surveys, the estimation of quality-adjusted life years, and in economic evaluations). The HUI3 system has eight dimensions: vision, hearing, speech, ambulation, dexterity, emotion, cognition, and pain, with five or six levels per dimension, varying from highly impaired to normal [15, 16]. The HUI3 score is expressed on a scale of 0 to 1: 0 represents a health state equivalent to death and 1 represents perfect health [15].

### Statistical analysis

Descriptive statistics (mean, standard deviation [SD] or percentage, as appropriate) were used for the demographic and clinical characteristics.

A ranked ordered logit model was used to estimate marginal utilities associated with each attribute, i.e., incremental utilities between different levels of a specific attribute. A random patient effect was entered into the model to account for the fact that the provided answers of a patient for different choice sets were not independent. The difference between coefficients corresponding to two levels of an attribute represented the marginal utility associated with a change from one level to the other. The cost attribute was entered as a continuous variable and a linear relationship between utility and cost was assumed. The model also included a dummy variable for the current inhaler, to capture a possible preference of patients for keeping their current inhaler, when all attributes are equal. The WTP for a given level of one attribute compared with another was estimated. Interactions between the attributes of asthma and COPD were tested. The model was adjusted with the most relevant covariates (e.g., age, gender, disease [asthma or COPD], income level, satisfaction level) in the data to test whether there is an effect of the covariate on the inhaler choice.

The WTP for a given attribute compared with another was estimated based on outcomes from the logistic model (the ratio of the marginal utility between those two attributes levels divided by the marginal utility associated with a loss of €1 [the opposite of the coefficient estimated for the cost attribute]). Credibility intervals around WTP were estimated using a Markov Chain Monte Carlo (MCMC) method. MCMC is a technique for estimating by simulation the expectation of a statistic in a complex model. Successive random selections form a Markov chain, the stationary distribution of which is the target distribution.

## Results

### Literature review

The literature review identified 16 studies in which patients were asked to express their preferences between different types of inhalers (DPIs, MDIs, SMIs, and nebulizers) using predefined inhaler characteristics [17–32]. None of the studies used patient interviews or focus groups to elicit important attributes specifically related to DPIs.

The literature review revealed no overall consensus on the importance of different inhaler attributes in relation to patient satisfaction across the studies. However, the most often cited inhaler characteristics were related to ease of use of the device (Table 1)**.**

Different terms were used across the different studies to describe relevant concepts for inhaler attributes. Table 1 summarises the different terms and the frequency these were used in the literature reviews.

### Patient focus groups

Patients focused mainly on inhaler characteristics related to ease of use, rather than performance, during the patient group discussions.

When asked (towards the end of the discussion) to select the most important inhaler attributes, patients most frequently mentioned the following: size of the dose counter, ability to keep the mouthpiece hygienic (can be washed or can be replaced), ergonomics of the inhaler mouthpiece, presence of a dosing feedback mechanism, ease with which a dose can be prepared, and low inspiratory flow requirements. Although, the mouthpiece of neither the Symbicort Turbuhaler nor Seretide Diskus can be washed or replaced, the hygiene feature was included in the focus group discussions as a factor to consider for future improvement.

Although the attributes selected by patients with asthma or COPD were generally similar, some differences between the two groups were reported: the size of the dose counter was selected as the most important feature of an inhaler by patients with COPD, while the ergonomics of the mouthpiece was selected as the most important feature of an inhaler by patients with asthma.

The outcomes from the literature review, expert interviews, and patient focus groups guided the development of a list of attributes and attribute levels, which were subsequently used in the quantitative study (Table 2). A final seven attributes each described by up to four levels were used in the main survey.

**Table 2:** Attributes and attribute levels

	Attributes	Levels
1	Ease of use/fool-proof priming	− 1 step − 2 to 3 steps − More than 4 steps to prepare your dose
2	Accurate and easy-to-read dose counter *Precise dose counter*	− Yes − No
3	Dose confirmation	− Taste of lactose − No taste of lactose
4	Hygiene of mouthpiece	− Mouthpiece can be washed, but not replaced − Mouthpiece can be replaced, but not washed − Mouthpiece can be cleaned with a dry cloth, but not washed or replaced
5	Flexibility of device handling *Orientation of device during inhalation process*	− Inhaler can be held in any position throughout inhalation process − Inhaler must be held in certain position throughout inhalation process
6	You can use the inhaler even when you have difficulties breathing in	− Yes − No
7	Costs associated with using the medication	− Current level of expenditure − Current level of expenditure + €3/month − Current level of expenditure + €6/month − Current level of expenditure + €10/month

### Pilot study

Twenty-five patients (10 with asthma, 10 with COPD and five with asthma/COPD) participated in the pilot Cognitive Debriefing interview to identify any potential areas of misunderstanding in the survey. Patients were asked to complete the survey in a ‘think aloud’ exercise. Another 17 patients answered the pilot survey and data were analysed to determine if attribute coefficients were all in the expected direction.

### Main DCE survey

A total 294 patients were included, 201 with asthma and 93 with COPD. Patient demographics and clinical characteristics are shown in Table 3.

**Table 3:** Patient demographics and clinical characteristics

Patient demographic characteristics		Asthma (*n* = 201)	COPD (*n* = 93)	Total (*n* = 294)
Age	Mean (SD)	40.30 (13.44)	48.48 (15.16)	42.89 (14.49)
Gender	Male	63 (31.34%)	42 (45.16%)	105 (35.71%)
	Female	138 (68.66%)	51 (54.84%)	189 (64.29%)
Current inhaler	Symbicort Turbuhaler	95 (47%)	44 (47%)	139 (47%)
	Seretide Diskus	106 (53%)	49 (53%)	155 (53%)
Revenue in last 12 months	<€22,500	97 (48.26%)	40 (43.01%)	137 (46.60%)
	€25,000–€34,999	39 (19.40%)	14 (15.05%)	53 (18.03%)
	€35,000–€49,999	30 (14.93%)	15 (16.13%)	45 (15.31%)
	€50,000–€74,999	15 (7.46%)	7 (7.53%)	22 (7.48%)
	€75,000+	2 (1.00%)	6 (6.45%)	8 (2.72%)
	No answer	18 (8.96%)	11 (11.83%)	29 (9.86%)
Costs associated with asthma and COPD treatment (€)/month	Mean (SD)	11.89 (21.93)	22.11 (37.37)	15.12 (28.10)
HUI3	Mean (SD)	0.71 (0.25)	0.54 (0.30)	0.66 (0.28)

*COPD* chronic obstructive pulmonary disease, *DPI* dry powder inhaler, *HUI3* health utilities index mark III, *SD* standard deviation

In both groups, the majority of patients were female (64% overall). The mean age of patients with COPD was higher compared with that of patients with asthma (48 vs. 40 years of age). Most patients (47% of total patients) reported annual earnings < €22,500. Seretide Diskus and Symbicort Turbuhaler were used by a similar proportion of patients in both groups. Approximately half of the patients (52% asthma; 46% COPD) reported that they were satisfied with their current inhaler. The reported spending on treatment was higher in patients with COPD compared to patients with asthma (€22.11 vs. €11.89 per month, respectively). The average HUI3 utility score was higher in patients with asthma (0.71) compared with patients with COPD (0.54).

### Patient preferences and willingness to pay for attributes

#### Preferences

**Fig. 1:**
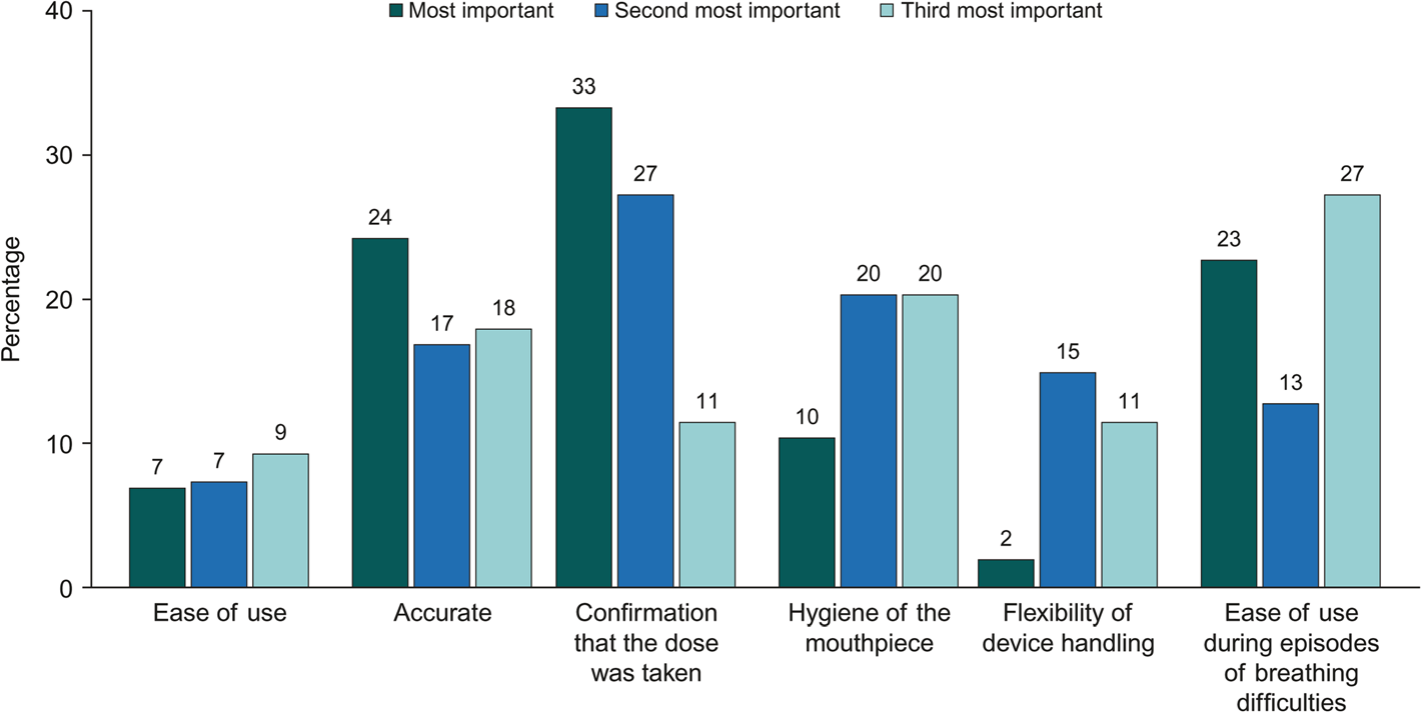
Three levels of importance of inhaler attributes ranked by patients with asthma

**Fig. 2:**
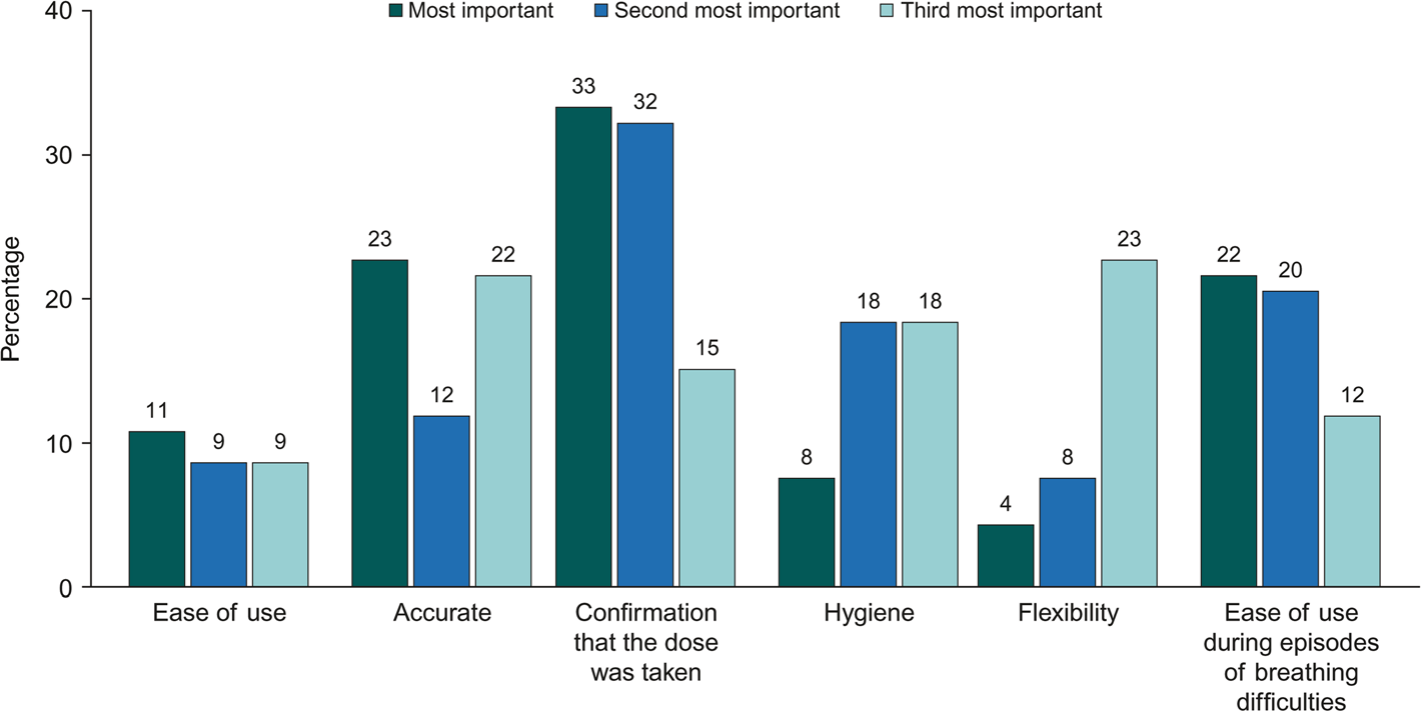
Three levels of importance of inhaler attributes ranked by patients with COPD. COPD, chronic obstructive pulmonary disease

Similar preferences were reported by patients with asthma and COPD when ranking the inhaler attributes they deemed most valuable (Fig. 1 and Fig. 2); these included: confirmation that the dose was taken (33% of patients with asthma/33% of patients with COPD), accurate and easy-to-read dose counter (24%/23%) and ease of use during episodes of breathing difficulties (23%/22%).

Analysis for interactions between disease (asthma and COPD) and attributes showed that the flexibility of handling and cost attributes were valued differently by asthma and COPD patients; therefore, separate models were estimated for asthma and COPD. Marginal utilities were statistically significant for all attributes except for hygiene (Table 4). Within the COPD group, 27% patients were <40 years of age (*n* = 25; Range 18–39 years Additional file 2: Table S2). Furthermore half of this younger sub-group of patients with COPD was <28 years-old. As the age of onset for COPD is typically 40 years or above, we performed an additional sub-analysis of patients who were ≥40 years (*n* = 68, Additional file 3: Table S3). When restricting the sample of COPD patients to those ≥40 years COPD, the ranking of attributes was unchanged, and marginal utilities were generally similar to those reported for the overall COPD population. (Additional file 4: Table S4).

**Table 4:** Results of ordered logit regression models for asthma and COPD samples

Parameter	Attribute level	Asthma			COPD		
		Estimate (SE)	*p*-value	Odds ratio	Estimate (SE)	*p*-value	Odds ratio
Ease of use	1 step	0.55 (0.081)	<0.0001	1.733	0.282 (0.118)	0.017	1.326
	2 or 3 steps	0.281 (0.069)	<0.0001	1.324	0.203 (0.1)	0.043	1.226
Accurate	Yes	0.377 (0.047)	<0.0001	1.458	0.419 (0.069)	<0.0001	1.52
Confirmation that the dose was taken	Taste of lactose	0.333 (0.045)	<0.0001	1.395	0.362 (0.066)	<0.0001	1.436
Hygiene	Mouthpiece can be replaced	–0.032 (0.063)	0.611	0.968	–0.028 (0.091)	0.757	0.972
	Mouthpiece can be washed	0.026 (0.063)	0.678	1.027	–0.073 (0.092)	0.427	0.929
Flexibility	Any position	0.164 (0.045)	0.0003	1.178	–0.03 (0.065)	0.646	0.97
Ease of use during episode of breathing difficulties	Yes	0.5 (0.051)	<0.0001	1.649	0.549 (0.075)	<0.0001	1.731
Monthly out-of-pocket money		–0.114 (0.012)	<0.0001	0.893	–0.067 (0.017)	<0.0001	0.936
Current inhaler	Yes	0.207 (0.086)	0.016	1.23	0.137 (0.126)	0.275	1.147

*COPD* chronic obstructive pulmonary disease, *SE* standard error

#### Willingness to pay

For most attributes, WTP estimates were higher for patients with COPD, attributed to a lower marginal utility for cost.

**Fig. 3:**
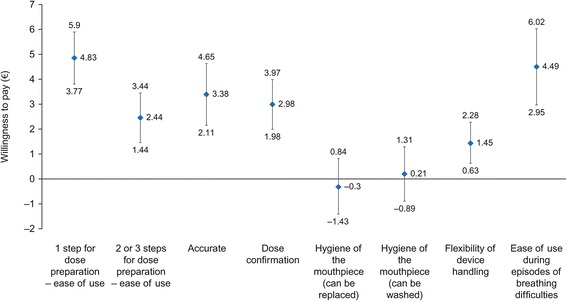
Willingness to pay in patients with asthma

Patients with asthma were willing to pay €4.83 per month (95% CI: €3.77–€5.90) for an inhaler that requires one step for dose preparation instead of four, €4.49 per month (95% CI: €2.95–€6.02) for an inhaler that could be used during episodes of breathing difficulties, €3.38 per month (95% CI: €2.11–€4.65) for an accurate and easy-to-read dose counter, €2.98 per month (95% CI: €1.98–€3.97) for an inhaler which provides confirmation that the dose has been taken, and €1.45 per month (95% CI: €0.63–€2.28) for flexibility of handling (Fig. 3).

**Fig. 4:**
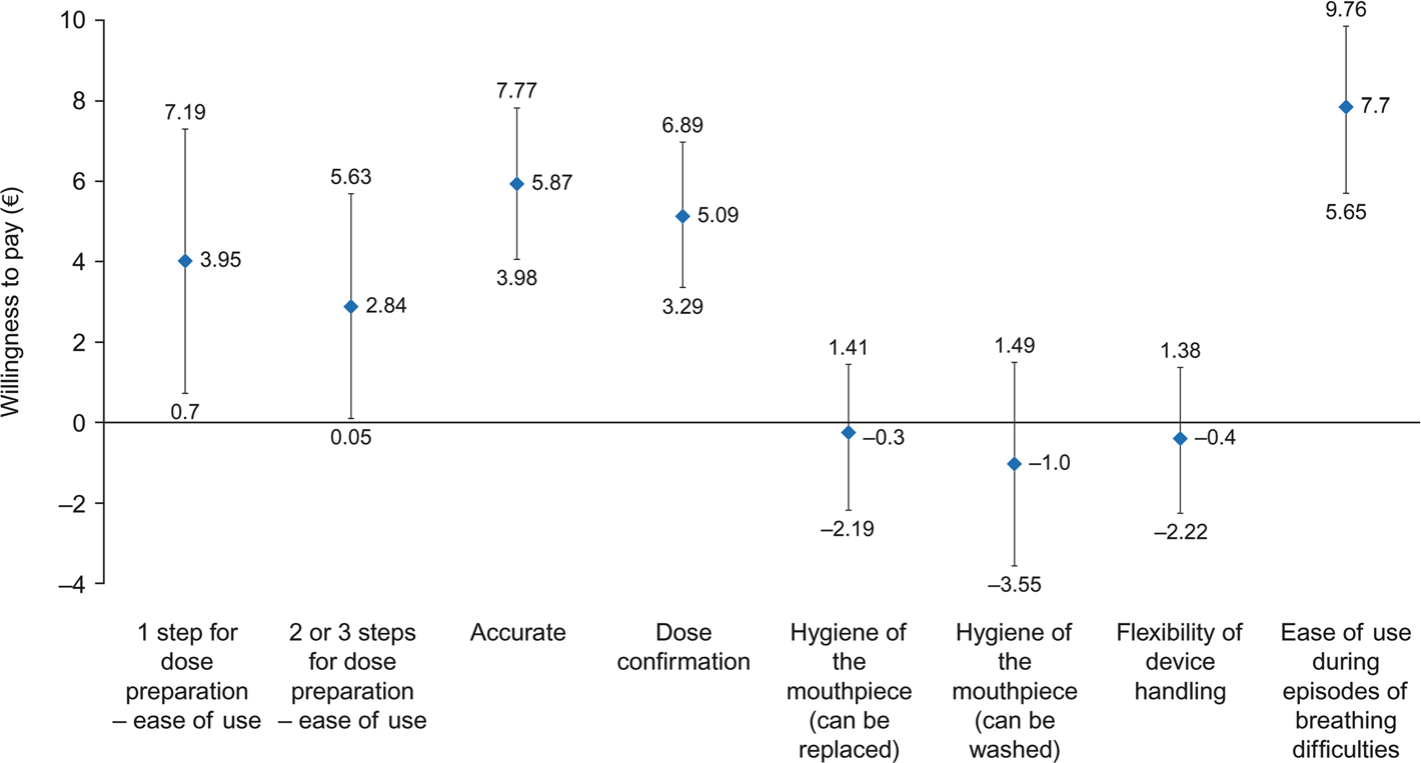
Willingness to pay in patients with COPD. COPD, chronic obstructive pulmonary disease

Patients with COPD were willing to pay €7.70 per month (95% CI: €5.65–€9.76) for an inhaler that could be used during episodes of breathing difficulties, €5.87 per month (95% CI: €3.98–€7.77) for an accurate dose counter, €5.09 per month (95% CI: €3.29–€ 6.89) for an inhaler which provides confirmation that the dose has been taken, and €3.95 per month (95% CI: €0.70–€7.19) for an inhaler which requires one step for dose preparation (Fig. 4).

#### Choice between current and new inhalers

Patients with asthma were more likely to choose their current inhaler over a new one (all attributes being equal [*p* = 0.016]). This was numerically similar for patients with COPD; however, the difference between patients who chose or declined their current inhaler was not statistically significant (Overall COPD group, *p* = 0.275 [Table 4]; ≥40 years COPD group, *p* = 0.537 [Additional file 4: Table S4]); the marginal utility for keeping the current inhaler is lower than the marginal utility of most DPI attributes.

A positive interaction between satisfaction with current inhaler and probability of choosing the current inhaler (*p* = 0.0005) was reported: patients who were satisfied with their current inhaler were more likely to choose it (all attributes being equal). Patients who were neither satisfied nor dissatisfied with their current inhaler were neither more likely nor less likely to choose their current inhaler over a new inhaler. Older patients, and those on high incomes, had a higher probability to choose their current inhaler over a new one (Table 5). The choice of the current inhaler was not related to gender.

**Table 5:** Results of models including interactions between current inhaler and patient characteristics

	Gender (ref = Male)	Age	Disease (ref = Asthma)	Income Income level (ref: <€25,000)	Satisfaction level^a^ (ref = Unsatisfied)
Ease of use	<0.0001	<0.0001	<0.0001	<0.0001	<0.0001
Accurate	<0.0001	<0.0001	<0.0001	<0.0001	<0.0001
Confirmation that the does was taken	<0.0001	<0.0001	<0.0001	<0.0001	<0.0001
Hygiene	0.8125	0.8222	0.823	0.7962	0.8241
Flexibility	0.0059	0.0056	0.0059	0.0059	0.0059
Use with difficulties breathing	<0.0001	<0.0001	<0.0001	<0.0001	<0.0001
Monthly out-of-pocket money	<0.0001	<0.0001	<0.0001	<0.0001	<0.0001
Current inhaler	0.0042	0.0004	0.0035	0.1962	0.5805
Current inhaler^a^ characteristic	0.0681	<0.0001	0.135	0.0052	0.0005

^a^Satisfaction levels were satisfied, neutral and unsatisfied assessing patient’s satisfaction with their current inhaler

## Discussion

This study was designed to elicit preferences for DPI attributes among patients with asthma and COPD in France.

The DCE included six device-related attributes and an out-of-pocket expense attribute. Preferences for these attributes were quantified in monetary terms. Five of the six device-related attributes were significant predictors for the choice of an inhaler, and thus had significant marginal utilities. Analyses for interactions between attributes and disease showed that patient preferences for DPI attributes were generally similar among patients with asthma or COPD, as marginal utilities did not differ significantly between groups for five attributes.

An easy-to-use inhaler device, and one that is preferred by patients (i.e. perceived positively), may facilitate correct handling of the inhaler and adherence to therapy, which could help in maintaining symptom control. The high rate of poor inhalation technique in real-life studies supports this hypothesis. It is reported that 20–82% of patients, depending on the type of inhaler and the method of assessment, do not use their DPIs correctly [4, 5, 8, 33, 34]. Moreover, 25% of patients have never received training for using their inhaler device [8], and >50% of patients have not had their inhaler technique checked within the last year [35]. Educating and training patients in the use of their inhalers is crucial for the success of the inhalation therapy and is now an integral part of the Global Initiative for Asthma (GINA) management strategy. GINA recommends that patients receive training in inhaler technique as a component of good clinical practice [36].

Patients with asthma placed the highest values on the ease of use and ability to use the inhaler during episodes of breathing difficulties, and for attributes that would provide patients with reassurances about the inhaled dose being taken, such as precise dose counters and dose confirmation mechanisms. Patients with COPD placed the highest values on the usability of the inhaler during episodes of breathing difficulties and, similar to patients with asthma, on attributes relating to the dose, including the accurate dose counter and dose-confirmation mechanism. Some differences between patients with asthma and those with COPD were also found in this study. Patients with asthma considered the attribute ‘flexibility of handling of an inhaler’ to be of significant value, whereas patients with COPD did not.

The orientation of the inhaler device and positioning during inhalation can affect the efficacy of dose delivery for some devices [3, 37]. A systematic review that evaluated the quality of inhalation technique with well-established DPIs in both adult and paediatric patients with asthma or COPD suggested that one of the most frequent errors with Turbuhaler is incorrect inhaler positioning [8]. In a recent study, patients using Diskus also displayed positioning error [37]. We expected that some of the older patients in this study would have difficulties in holding their inhaler in the right position, for example, because of age-related osteoarthritis or neurologic conditions, and that they would place a high value on this particular attribute. However, our data showed that this was not the case, even though 13% of patients with COPD had dexterity problems and might require support in operating their inhaler. It could be argued that patients were not aware that they were not holding the inhaler correctly; as such, they did not place value on this attribute.

Although a source of difficulties with older established DPIs, device positioning error may have been improved in newer DPIs; for instance, a report on the Spiromax device published in 2015 demonstrated that despite different orientations (45° or 90° tilt) under controlled laboratory conditions dosing consistency was maintained [38]. Further studies are needed to assess orientation-dependent dosing changes in real world scenario for Spiromax, as well as for comparisons with dosing deviations of Turbuhaler and Diskus.

The finding that younger patients were more likely to choose a new inhaler is consistent with our expectations that older patients may be more conservative and/or less demanding, and more likely to prefer their current inhaler over a new one. In particular, the interaction between satisfaction with the current inhaler and the patient’s preference with the current inhaler was positive.

The finding that patients’ value the ‘ease of use’ attribute is consistent with a previous study by Kawata et al. [39], which evaluated patient preference and WTP for attributes of maintenance medication for COPD. Outcomes from this study showed that patients with COPD were willing to pay $16 per month for an inhaler that is ‘quick and easy to use’, compared with an inhaler that is ‘more difficult and time-consuming’ to use. Patients’ perception of ‘ease of use’ as defined by Kawata et al. [39] probably encompasses several of the attributes included in our study. Also, the estimates of WTP in our study were comparable to the values previously published.

In this study population, the proportion of women was higher than men for both asthma and COPD groups. The asthma group could be considered to be representative of the French population of patients with asthma. The reported average age of patients with asthma in France was 37 years in 2006, and the prevalence of the disease is higher in women compared with men [40]. However, the prevalence of COPD is usually higher in men compared with women [41]. The use of a web-based sample to enrol patients could have played a role in this discrepancy, as women are more likely to participate in online surveys compared with men [42]. Patients with asthma were younger and in better health compared with patients with COPD, demonstrated by a higher HUI3 utility score.

There are some limitations to this study: some counterintuitive interactions are noted in the sections above. First, as discussed earlier, 27% of the patients with COPD were under 40 years-old, half of whom were younger than 28 years, which suggests that the diagnosis of COPD was mistakenly reported by some patients. The device attribute data collected from these younger patients with COPD may have confounded the overall COPD group analysis. Therefore, we performed a sub-analysis of patients who were 40 years-old or older and found similar attribute outcomes between the ≥40 year-old patients and the overall COPD study population, suggesting that the data derived from the younger patients did not skew the overall outcome. Second, this study did not assess the aftertaste of the medication used and only accounted for taste of lactose as part of the dose-confirmation attribute. The formulation of the medication has been shown to affect preference. In a multicentre, randomised, open-label, crossover study with two 4-week evaluation periods, which compared patient preference and ease of teaching correct inhaler technique for Pulmicort® Turbuhaler versus pMDIs, 64% of patients preferred Pulmicort Turbuhaler over pMDIs since it was not associated with a ‘bad’ aftertaste [32]. Third, the ergonomics of the mouthpiece was not included in the DCE attributes (although it was found to be an important attribute in focus groups) due to the exclusion of attributes that could not be described in an objective way. Finally, the number of attributes included in the DCE was kept to a concise number in order to simplify the selection of attributes that are deemed most important by patients. This selection, although necessary for this study, could be viewed as somehow biased due to the subjective nature of preselecting attributes by the study team.

## Conclusion

In conclusion, findings from this study suggest that patients with asthma and COPD share similar views with regards to inhaler attributes that they value most in a device and would change their inhaler if they were offered the option of a new inhaler with improved characteristics. Patients placed highest values on attributes related to ease of use and ability to use the inhaler during episodes of breathing difficulties.

## Additional files

Additional file 1: Table S1. Literature search keywords used to identify inhaler attributes in studies assessing treatment satisfaction among patients with asthma and COPD. (DOCX 14 kb)

Additional file 2: Table S2. Age distribution of under 40 years old patients with COPD. (DOCX 13 kb)

Additional file 3: Table S3. Patient demographics and clinical characteristics for COPD patients aged over 40 years old. (DOCX 14 kb)

Additional file 4: Table S4. Results of ordered logit regression models for COPD patients aged over 40 years old sample. (DOCX 14 kb)

## Abbreviations

BA-MDI: Breath-actuated metered-dose inhalers
CI: Confidence interval
COPD: Chronic obstructive pulmonary disease
DCE: Discrete choice experiment
DPI: Dry powder inhaler
FrF: Fractional factorial
GINA: Global initiative for asthma
HUI3: Health utilities index mark III
MCMC: Markov Chain Monte Carlo
NHS: National Health Service
pMDI: Pressurised metered-dose inhaler
SD: Standard deviation
SE: Standard error
SMI: Soft mist inhalers
WTP: Willingness to pay
