# Supplementary material for: Patient preferences for dry powder inhaler attributes in asthma and chronic obstructive pulmonary disease in France: a discrete choice experiment
Source: BMC Pulm Med. 2017 Jul 6;17:99. doi: 10.1186/s12890-017-0439-x (PMC5501405; doi:10.1186/s12890-017-0439-x)
Supplement: Supplementary file 1 — Literature search keywords used to identify inhaler attributes in studies assessing treatment satisfaction among patients with asthma and COPD. (DOCX 14 kb) [file 12890_2017_439_MOESM1_ESM.docx]

**Additional file 1: Table S1.** Literature search keywords used to identify inhaler attributes in studies assessing treatment satisfaction among patients with asthma and COPD.

|  | **Search Terms** | **# of records identified** |
| --- | --- | --- |
| 1 | Asthma.ab,ti. | 106910 |
| 2 | COPD.ab,ti. | 24010 |
| 3 | Chronic obstructive pulmonary disease.ab,ti. | 25951 |
| 4 | 1 or 2 or 3 | 135661 |
| 5 | Inhaler$.ab,ti. | 6073 |
| 6 | 4 and 5 | 3263 |
| 7 | Discrete choice experiment or DCE {Including Related Terms} | 17057 |
| 8 | Patient$ preference$ {Including Related Terms} | 8024 |
| 9 | Preference$ {Including Related Terms} | 5980 |
| 10 | Satisfaction$.ab,ti. | 73589 |
| 11 | Focus groups$.ab,ti. | 20673 |
| 12 | (interview or qualitative).ab,ti. | 202247 |
| 13 | or/7-11 | 122073 |
| 14 | 6 and 13 | 129 |
| 15 | Limit 14 to (English language and humans and yr=”2003-current”) | 71 |

Database: Medline^®^ (OVID) In-Process & Other Non-Indexed Citations; Medline^®^ (OVID) Daily; Medline^®^ (OVID) and Medline^®^ (OVID) <1946 to Present>

Search executed on January 14, 2014

COPD, chronic obstructive pulmonary disease
