# Supplementary material for: Patient preferences for dry powder inhaler attributes in asthma and chronic obstructive pulmonary disease in France: a discrete choice experiment
Source: BMC Pulm Med. 2017 Jul 6;17:99. doi: 10.1186/s12890-017-0439-x (PMC5501405; doi:10.1186/s12890-017-0439-x)
Supplement: Supplementary file 2 — Age distribution of under 40 years old patients with COPD. (DOCX 13 kb) [file 12890_2017_439_MOESM2_ESM.docx]

**Additional file 2: Table S2.** Age distribution of under 40 years old patients with COPD

|  |  | **COPD under 40 years old**  ***n* = 25** |
| --- | --- | --- |
| **Age** | Mean (SD;SE) | 27.96 (6.27 ; 1.25) |
|  | Min-Max | [18 ; 39] |
|  | Median | 28 |

COPD, chronic obstructive pulmonary disease; SE, standard error; SD, standard deviation
