# Supplementary material for: Patient preferences for dry powder inhaler attributes in asthma and chronic obstructive pulmonary disease in France: a discrete choice experiment
Source: BMC Pulm Med. 2017 Jul 6;17:99. doi: 10.1186/s12890-017-0439-x (PMC5501405; doi:10.1186/s12890-017-0439-x)
Supplement: Supplementary file 3 — Patient demographics and clinical characteristics for COPD patients aged over 40 years old. (DOCX 14 kb) [file 12890_2017_439_MOESM3_ESM.docx]

**Additional file 3: Table S3.** Patient demographics and clinical characteristics for COPD patients aged over 40 years old

| **Patient demographic characteristics** |  | **COPD ≥ 40 years old**  **(*n* = 68)** |
| --- | --- | --- |
| **Age** | Mean (SD) | 56.03 (9.29) |
| **Gender** | Male | 29 (42.65%) |
|  | Female | 39 (57.35%) |
| **Current inhaler** | Symbicort Turbuhaler | 34 (50.00%) |
|  | Seretide Diskus | 34 (50.00%) |
| **Total use of DPI in years** | <5 years | 21 (30.88%) |
|  | 5–10 years | 16 (23.53%) |
|  | 10–15 years | 8 (11.76%) |
|  | >15 years | 23 (33.82%) |
| **Revenue in last 12 months** | <€22,500 | 27 (39.71%) |
|  | €25,000–€34,999 | 14 (20.59%) |
|  | €35,000–€49,999 | 3 (4.41%) |
|  | €50,000–€74,999 | 12 (17.65%) |
|  | €75,000+ | 3 (4.41%) |
|  | No answer | 9 (13.24%) |
| **Costs  associated with asthma and COPD treatment (€)/month** | Mean (SD) | 12.62 (19.01) |

COPD, chronic obstructive pulmonary disease; DPI, dry powder inhaler; SD, standard deviation
