# Supplementary material for: Patient preferences for dry powder inhaler attributes in asthma and chronic obstructive pulmonary disease in France: a discrete choice experiment
Source: BMC Pulm Med. 2017 Jul 6;17:99. doi: 10.1186/s12890-017-0439-x (PMC5501405; doi:10.1186/s12890-017-0439-x)
Supplement: Supplementary file 4 — Results of ordered logit regression models for COPD patients aged over 40 years old sample. (DOCX 14 kb) [file 12890_2017_439_MOESM4_ESM.docx]

**Additional file 4: Table S4.** Results of ordered logit regression models for COPD patients aged over 40 years old sample

| **Parameter** | **Attribute level** | **COPD ≥ 40 years old** | | | |
| --- | --- | --- | --- | --- | --- |
|  |  | **Estimate (SE)** | **p-value** | **Odds ratio** | **WTP (95% CI)** |
| **Ease of use** | 1 step | 0.398 (0.14) | 0.0045 | 1.489 | 3.46 (1.5 ; 5.42 ) |
|  | 2 or 3 steps | 0.208 (0.121) | 0.0845 | 1.231 | 1.73 (-0.21 ; 3.68 ) |
| **Accurate** | Yes | 0.463 (0.084) | <.0001 | 1.588 | 4.38 (1.59 ; 7.16 ) |
| **Confirmation that the does was taken** | Taste of lactose | 0.415 (0.079) | <.0001 | 1.514 | 3.86 (1.76 ; 5.96 ) |
| **Hygiene** | Mouthpiece can be replaced | -0.047 (0.11) | 0.6702 | 0.954 | -0.49 (-2.57 ; 1.6 ) |
|  | Mouthpiece can be washed | -0.146 (0.109) | 0.1801 | 0.864 | -1.44 (-3.74 ; 0.87 ) |
| **Flexibility** | Any position | -0.033 (0.079) | 0.6733 | 0.967 | -0.33 (-1.82 ; 1.15 ) |
| **Ease of use during episode of breathing difficulties** | Yes | 0.598 (0.089) | <.0001 | 1.818 | 5.65 (2.34 ; 8.95 ) |
| **Current inhaler** | Yes | 0.091 (0.148) | 0.5365 | 1.096 | 1.04 (-1.93 ; 4.01 ) |
| **Monthly out of pocket money** | | -0.112 (0.02) | <.0001 | 0.894 | - |

CI, confidence interval; COPD, chronic obstructive pulmonary disease; SE, standard error; WTP, willingness to pay
